# Engagement of ethics and regulatory authorities on human infection studies: Proceedings of an engagement workshop in Zambia

**DOI:** 10.12688/wellcomeopenres.16432.1

**Published:** 2021-02-12

**Authors:** Evelyn Muleba Kunda-Ng'andu, Michelo Simuyandi, Melissa Kapulu, Masuzyo Chirwa-Chobe, Hope Mwanyungwi-Chinganya, Stanley Mwale, Roma Chilengi, Anjali Sharma

**Affiliations:** 1 Research Department, The centre for Infectious Disease Research in Zambia, Lusaka, Zambia, 10101, Zambia; 2 Biosciences, KEMRI-Wellcome trust research Programme, Kilifi, Kenya; 3 Nuffield Department of Medicine, Centre for Tropical Medicine and Global Health, University of Oxford, Oxford, UK

**Keywords:** Human Infection Challenge Studies, HIC, Challenge agent, Vaccine, Compensation, Zambia, Resource limited setting, informed consent, Rotavirus, Vibrio Cholerae, Shigella, Salmonella Typhi

## Abstract

Human infection studies (HIS) have generally been used as a tool in the pathway for vaccine development in high income settings. Over the last decade, this model has been implemented in LMICs with the aim of accelerating development of next generation vaccines that would perform better in these settings. However, in most LMICs, the ethics and regulatory framework for the conduct of these studies are not in place. In Zambia, these studies are yet to be conducted and thus we conducted a stakeholder engagement workshop in October 2019. We engaged with bioethicists, regulatory authority, and scientists from within Zambia and other African countries to anticipate and address foreseeable ethical and regulatory issues when conducting HIS in Zambia for the first time. The workshop largely focused on sensitizing the stakeholders on the benefits of these studies with the following main points for consideration on the implementation of these studies in Zambia: need for in-country legal framework and guidelines; need for adequate informed consent based on comprehensive understanding of the concept of HIS and study requirements; and requirements for heightened vigilance to assure participant safety including good ethical and clinical practice with regulatory, ethical, data safety, and community oversight. Additionally, the workshop emphasized the need for rigorous health screening prior to enrolment; suitable infrastructure for containment; and personnel to provide appropriate treatment including emergency resuscitation and evacuation if indicated. Specific recommendations included compensation for burden of participation; access to care and provision for study related injury (e.g. no-fault insurance); and withdrawal and exit procedures to preserve individual and community safety. Finally, the meeting concluded that researchers should actively engage key gate keepers including civic leaders such as parliamentarians, universities, researchers, potential participants and laypersons to avoid circulation of misinformation.

## List of abbreviations

CIDRZ      Centre for infectious disease research in Zambia

DSMB      Data Safety Monitoring Board

GCP      Good Clinical Practice

GMO      Genetic Modified Organism

GMP      Good Manufacturing Practice

HIS      Human Infection Studies

ICF      Informed Consent Form

IP        Investigational Product

KEMRI      Kenya Medical Research Institute

LMICs      Low and Middle Income Countries

NBA      National Biosafety Authority

NHRA      National Health Research Authority

REGs      Regulatory authorities

WHO      World Health Organization

ZAMRA      Zambia Medicines Regulatory Authority

## Disclaimer

The views expressed in this article are those of the author(s). Publication in Wellcome Open Research does not imply endorsement by Wellcome

## Introduction

### Setting up for enteric human infection studies in Zambia

It is evident that low- and middle-income countries (LMICs) carry the higher burden of infectious disease in comparison to high income countries ^1– 3^. Most of these infectious diseases are preventable. However, children in LMICs, who are the most affected succumb to these diseases ^1^ including children in Zambia ^4^, partly because that life-saving interventions have historically been developed in high-income settings ^5, 6^.

Vaccines are among the major cost-effective interventions in global public health with vast social and economic benefits ^7^. The lower vaccine efficacy and effectiveness observed in LMICs ^1, 8^ speaks to the need for next generation vaccines developed in these endemic settings to supplement existing vaccines. The urgent need to address this health disparity has led to the introduction of human infection studies (HIS) in LMICs. HIS can accelerate the pace of vaccine discovery and testing by providing an early opportunity to evaluate efficacy, circumventing the difficulties, cost, time and unpredictability of large field trials ^9^. HIS involve deliberate or intentional infection by administration of pathogens to healthy and consenting research participants under controlled conditions with the aim of: (i) evaluating candidate vaccines and therapeutics; (ii) gaining insight into natural infections; and (iii) developing a model of infection ^6, 10^. HIC also facilitate detailed understanding of infectious disease pathophysiology, immunological defence mechanisms, host pathogen interactions ^11^ and provide opportunities to identify useful correlates of vaccine protection ^12^. Historically, the majority of HIS studies have been conducted in high-income, non-endemic settings, which has raised calls to establish HIS models in LMICs so that the findings can be relevant to the population at risk ^13^.

The Centre for Infectious Disease Research in Zambia (CIDRZ) is poised to establish enteric disease HIS models targeting Rotavirus, *Shigella* and *Salmonella*; with potential to extend to other pathogens including *Enterotoxigenic Escherichia coli* (ETEC) and *Vibrio cholerae*. As HIS are new to Zambia, we aimed to create a platform upon which we could initiate the HIS conversation and contribute to strengthening capacity of ethical and regulatory bodies in governing HIS through an ethical and regulatory framework relevant for Zambia and LMICs more widely. Hence, we conducted an engagement workshop in order to: 1) understand views, expectations, and experiences of ethical and regulatory bodies, and other stakeholders involved in HIS in the region; 2) identify core ethical issues for HIS implementation in other LMICs and their implications for HIS in Zambia; and 3) develop modalities to address these issues from lessons learnt. Similar workshops have been conducted in Malawi ^14^, India ^15^, Uganda ^16^, with the main outcomes presented in Table 1. While these publications helped guide the content matter of our workshop, they did not provide any information on how such engagement workshops should be structured to hold the attention of experts in the field and to facilitate a process of mutual learning among stakeholders with different interests and areas of expertise. Given this, in addition to reporting the outcomes and perspectives of the workshop, we detail our methodology and the type of information they were able to elicit to guide future workshops.

**Table 1.** Summary of workshop efforts and outcomes on human infection studies (HIS) in low- and middle-income countries (LMIC) settings.

Workshop/meeting considerations	Host/country	What is stood out to the Zambian meeting
✓ discussed the benefits and barriers to HIC studies ✓ discussed the scientific and public health value of HIC ✓ discussed implementation challenges and recommended preparatory steps ✓ Considered research governance issues that need to be addressed including - the readiness of scientific and clinical stakeholders in the respective countries - advisability of challenge model implementation	Malawi, India, Uganda, Zambia, Vietnam, Kenya	Zambia through CIDRZ is willing to build capacity in producing our own challenge agent The discussions of the ethical implications of continuing to monitor the participant after withdrawing from a study to ensure they cleared of infection took centre stage. It was resolved that it was more ethical to ensure the participant is cleared of infection for their own safety and that of the community they come from.
**Potential benefits of HIC discussed included** ✓ Building local capacity in: clinical facilities; laboratory diagnostics; experimental medicine; clinical governance; and regulatory confidence. ✓ Opportunity to construct regulatory frameworks to suit local needs rather than adopting one from theEU/US. ✓ Improving science capacity through work-based training and mentorship of local scientists. ✓ Understanding key scientific questions relevant to public health in Malawi, including the effect of genetics, endemic infectious disease and co-infections, immune status, and environmental factors	Malawi, Zambia, Vietnam	
✓ These unique combinations of effects cannot be correctly understood in a model run elsewhere ✓ Accelerating or streamlining vaccines/treatment relevant to the national health burden.	Malawi	
✓ Participants were to be isolated from days ranging between 21 for typhoid and 28 for a Malaria CHIM	Zambia, Malawi	
✓ Participants were to be let go only when they were cleared of the infection	India, Zambia	
✓ Discussed the potential risks of using Genetically modified organisms (GMO) with issues raised concerning the mutation of the challenge agent beyond the controlled strain	Zambia, India Vietnam and Zambia	
**Benefits** ✓ improved vaccine effectiveness ✓ host country capacity development in clinical, laboratory and governance domains	Vietnam, Zambia, Malawi, Kenya	With regard to infrastructure, it was noted that CIDRZ is building infrastructure that will be specifically used for HIC studies Resolved that with compensation, the reimbursement model will be most appropriate With regard to GMOs, our meeting resolved that there is need to sensitize the communities on what GMO really are and the benefits that they have.
**Barriers** ✓ Acceptability, safety, and regulatory issues. ✓ The report suggests a framework by which ethical, laboratory, scientific and governance issues may be addressed by investigators considering or planning HIC in LMIC. **Challenges** ✓ Current infrastructure and clinical facilities may not be ready for CHIM (specifically, monitoring and supporting adverse events e.g. on intensive care units). ✓ Inherent vulnerabilities may hamper fully informed consent in the local context (languages, assessment understanding, participant criteria suitability, cultural family/group consenting).	Vietnam, Zambia, Malawi, Kenya	
✓ Poor community hygiene and sanitation infrastructure could prevent effective control measures (e.g. typhoid in out-patient settings). ✓ Production of challenge strain locally may have quality assessment issues, but “international” strains may be less relevant	Malawi, Zambia, Uganda	
✓ Resulting long supply chains need careful management. ✓ Consensus on appropriate compensation for monetary and opportunity costs are lacking: the balance between appropriate recompense and incentivisation is difficult and locally variable.	Vietnam, Zambia, Malawi, Kenya Zambia,	
✓ Not yet hosted Phase 1 studies, or those including healthy volunteers who are expected to become symptomatic due to the research protocol;	Malawi	
✓ there is a relative shortage of skills and experience were identified with regards to the technical procedures (around importation and, or, local production of challenge and related risk assessment); ( this is common to all meetings) ✓ community engagement and participant recruitment (around ensuring awareness and full understanding of study procedures ✓ and management of the potential for natural exposure during challenge experiments); ✓ and ethical as well as regulatory processes (around development of regulatory capacity, documentation and risk assessments)	Malawi, Uganda, India, Vietnam	
**Scientific reasons** to consider further CHIM in LMIC therefore include: ✓ the need to focus testing of late stage novel vaccines; ✓ the record of CHIM success in enteric vaccine ✓ malaria vaccine development ✓ the potential for accelerated development of as yet undiscovered vaccines.	Malawi	
✓ Attended by Regulators, community members, researchers and policy-makers ✓ looked at risk assessment; development of infrastructure and technical capacity to produce the infectious challenge material in country ✓ community engagement from Parliamentary to grass-roots level; ✓ pilot studies to establish approaches to assuring fully informed consent and true voluntariness	Vietnam, Zambia, Malawi, Kenya Uganda, Zambia	
✓ and strategies for selection of volunteers who can avoid natural infection during the 12-week HIC ✓ the building of regulatory capacity; and the development of study protocols and a product dossier in close consultation with ethical and regulatory partners.	Uganda Uganda, Zambia	
**Recommendations** ✓ on completion, the protocol and product dossier be reviewed for approval in a joint meeting combining ethical, regulatory and environment management authorities.	Uganda, Zambia	Four models of compassion were discussed in detail and the need to balance the risks and benefits in considerations for compensation It was noted in our meeting that insurance policies are a vital process of our clinical trials and that in a HIC it is even more necessary to ensure participants are on an insurance plan Unlike in India, we discussed the need to explore how HIC fits in existing regulatory frameworks It was agreed in our meeting that volunteers needed to be screened carefully to ensure they are suitable for the challenge. It was also noted that a system to track participants was be put in place to ensure they are not in several trials at once
✓ Most importantly, representatives of schistosomiasis-affected communities emphasized the urgent need for an effective vaccine and urged the research community not to delay in the development process.	Uganda	
✓ There is need to involve opinion leaders, including members of Parliament such as the Parliamentary Committee on Health, and Resident District Commissioners and District Health Officers of the participating districts, as well as local council leaders, in order to prevent circulation of misinformation about the work.	Uganda, Zambia, Malawi	
✓ It was agreed that volunteers would be expected to inform their next of kin about their participation and that contact details for the next of kin must be provided in case of emergency.	Uganda, Zambia, Vietnam	
✓ It is generally considered that participants should not be compensated for risk, since this could be interpreted as an undue inducement to take risks (similar to our discussion findings)	Uganda, Zambia, Vietnam	
✓ well placed to undertake as HIC because they have:	Uganda,	
(1) good laboratory facilities and expertise in maintaining the S. mansoni laboratory life-cycle, as well as in molecular and immunological work. (2) strong experience of community engagement, and expertise in clinical trials, complemented by an open and engaged ethical and regulatory environment	Uganda	
✓ The regulatory framework for HIC studies is complex and different from drug trials.	India	
✓ Insurance is an important factor to be considered. Long term health insurance coverage for participants may be needed.	Zambia, India, Vietnam	
✓ There is need for a multi-disciplinary ethics committee to review CHIM studies with specific domain expertise and training, and for an appropriate Government regulatory body.	Zambia, India	
✓ Regulatory and ethical frameworks must be developed in consultation with the public and the various stakeholders; with transparency and due diligence.		
✓ Desk research along with qualitative data on perceptions of various stakeholders will provide the evidence base for regulations ✓ A ‘regulatory sandbox’ approach was described, where existing legal frameworks can be put on hold and regulations can evolve incrementally by observation of the process in (HIC) studies conducted at one or two carefully selected institutions with oversight and monitoring	India	
**Key ethical issues** that were discussed included: ✓ ‘informed and understood consent’ ✓ Questions deliberated included: Is there data on how many times a participant can volunteer for CHIMs? What is the basis for such a moratorium? Is there a need for a central designated Ethics Committee for CHIMs at the country level?	Zambia	
✓ Any SAE emerging from a CHIM study should be evaluated differently from other clinical trials. Hence, specific guidance is needed for IECs monitoring the conduct CHIMs including SAEs and treatment of possible side effects of therapeutics involved.	India	
✓ There is need to go for pathogens that can be controlled and those that have a cure	India, Malawi, Zambia	
✓ Media recognized as a major player and stakeholder in the conduct of HIC	Vietnam, Zambia, Kenya	
✓ Students as the best participants	Zambia, Vietnam	

Note: References of meeting reports from Vietnam, Malawi, India, and Uganda whilst other country studies are personal communication from meeting participants (Kenya); CHIM, Controlled Human Infection Model; SAE, US, EU, IEC, ( 3, 14– 16)

### Considerations in planning the workshop

The requirements of ethical and regulatory environment governing the conduct of research and clinical trials in Zambia drove the decision of who to invite for this meeting. First, ethical committees (i.e., University of Zambia Biomedical Research Ethics Committee, Tropical Diseases Research Centre Research Ethics Committee) review study protocols, followed by regulatory authorities (i.e., Zambia Medicines Regulatory Authority and/or National Biosafety Authority), after which the National Heath Research Authority authorises implementation. Thus, representatives from these organizations were critical to achieving the aims of this workshop. Other considerations included gathering experiential knowledge and insights from international (regional) ethics committees and regulators who had reviewed HIS protocols and regulated their implementation as well as collecting the perception and perspectives of local researchers who are knowledgeable about the context and field realities in Zambia. This combination of local and regional knowledge and experience helped highlight issues that maybe unique to Zambia and to HIS implementation respectively.

In order to reduce the burden of participation for attendees while ensuring that they remained engaged and owned the whole process, we used a combination of methods that provided structure to guide proceedings but also the flexibility to allow attendees to share personal stories on key aspects of concern to HIS. Distribution of reading materials ahead of the meeting and didactic sessions aimed to create a common understanding on the current information on HIS implementation and ethical and regulatory issues arising. Participatory methods, empathy and journey mapping ^17^ helped promote discussions among small specialist and multi-disciplinary groups. Open and debriefing sessions helped collect other views outside the set agenda which might be critical to scientific and ethical conduct of HIS.

The workshop was set in Siavonga, a Zambian small and quiet town situated 200 kilometres away from the busy city of Lusaka which offered the delegates a serene environment for focused deep conversations.

We used didactic presentations to introduce themes and topics, provide background and rationale for HIS, and to frame the key issues for discussion. Presentations were delivered within 20 minutes in keeping with the expected attention span among adults ^18^. The themes and topics covered included: historical perspective and scientific rationale, the benefits and need for HIS in Zambia, methodological issues that can impact implementation and outcome of HIS, an overview of planned HIS in Zambia, defining risks and burdens of participation with application to HIS in Zambia, ethical considerations particularly for compensation supported by experiences shared by Kenyan and Malawian ethical review board members, scientific overview and responsibility of the scientists in the development of a challenge agent, and a funders’ perspective regarding the global agenda and direction for HIS in LMICs. Open questions and discussions concluded these sessions.

We employed small group discussions to deliberate on specific topics deemed critical. These groups were organised by

1. Specialisations (i.e. regulators, ethicists, community engagement and scientists) for the discussions around methodological considerations for HIS in Zambia.
2. Multidisciplinary for the discussions on: a) risk and burden of participants including through participant empathy and journey mapping; and b) benefits of HIS and participant safety.

Plenary discussions were held after each small group session, where group representatives presented their main discussion points to the meeting followed by questions and answers.

### Summary of the considerations for HIS in Zambia

A total of 29 participants drawn from four different countries namely, Malawi, Kenya, UK, and Zambia attended. These participants were drawn from 4 specialisations which included ethics, community engagement, social science, funder regulatory and clinical trial conduct as shown in Table 2.

**Table 2.** Workshop attendees.

Specialization	Zambia	Malawi	Kenya	UK	Total
Research ethics	5	3	1		9
Regulation on importation and biosafety	6		1		7
Clinical trials	4		1		5
Community engagement	5				5
Social science	2				2
Funder				1	1
**Total**	22	3	3	1	29

The didactic sessions and the group discussions yielded key points to be considered as Zambia prepares to conduct HIS. These key considerations included but were not limited to: (i) legal and regulatory provisions and guidelines; (ii) engagement strategy to introduce HIS; (iii) compensation models to be considered for HIS in Zambia; (iv) identifying population target participants for HIS; (v) risk-benefit ratio coupled with human subject protection; and (vi) balance of adequate information during the consenting process against burden of participation (see Table 3).

**Table 3.** Compensation models.

Model	Its code of belief
Market Model	• provides incentives to facilitate recruitment • openly stated in recruitment advertisements in developed countries • Escalate payment to meet recruitment
Wage Model	• compensates for time, effort and discomfort by standardizing wage-like payments irrespective of study completion to remove undue influence,
Reimbursement Model	• used to meet out-of-pocket expenses
Appreciation Model	• is a token given for participation in research

*Adapted from references ^20, 21^

### Legal provisions and regulatory guidelines

Research in Zambia is generally guided by the laws of the land which include the National Health Research Act No. 2 of 2013, Zambia Medicines and Allied Substances Act No. 13 of 2013, the Zambia National Biosafety Act No. 10 of 2007 and the Science and Technology Act No. 26 of 2007.

During the meeting, regulatory authorities highlighted the need to further investigate the laws under which the conduct of HIS would be permissible in Zambia and the process of reviewing HIS applications. They undertook to examine whether importation could be regulated using the World Health Organisation (WHO) categorisation of challenge agents as vaccines and HIS as a clinical trial ^17, 19^. They considered the workshop engagement as opportune and timely for their submission of amendments to the current Act to the Ministry of Justice that could propose relevant clauses to cover the conduct of HIS. They discussed another avenue for legal guidance by way of a specific Statutory Instrument which could be proposed by the Ministry of Health specifically covering the conduct of HIS in Zambia.

The National Biosafety Authority (NBA) on the other hand has the mandate to oversee biosafety and regulate importation and use of all products containing any genetically modified organisms (GMOs) and their products. Section 10 of the National Biosafety Act provides that “ *A person shall not research on, develop, produce, import, export, transit, carry out any contained use, release or place on the market any genetically modified organism or any product of a genetically modified organism or deal in any manner with any genetically modified organism or a product of a genetically modified organism without the prior approval of the Authority” ^22^.*

During the meeting, it was suggested that while the NBA act does not cover for the contained use of challenge agents or strains for HIS, an application can be considered based on scientific merit. It was further noted that, indeed challenge agents for use in HIS will likely meet the category of products overseen by NBA and will require their review and approval before use of the agents in Zambia. Of note, the NBA is required to ensure all such products are notified to the public through open media or press for at least 30 days before their intended importation or use. It was thus suggested that it would be critical to include the need for prior announcement in the media and/or press in the regulatory planning for HIS in Zambia. 

### Engagement strategy to introduce HIS

Given that HIS is a new concept in Zambia, the meeting agreed that an engagement strategy must be put in place to sensitise and engage various stakeholders over and above the regular processes used in clinical studies. Correct messaging and delivery through key gatekeepers such as political and civic leadership including the parliamentary committee on health, lecturers, editors for various forms of media houses, advocates, and religious leaders could lead to authentic community and participant engagement in HIS ^23^ which is critical to gaining permissions, approvals and legitimacy for any planned study ^24^. Having informed and influential stakeholders both in formal and communal structures are essential to safeguard study volunteers and to stand in defence in any event of issues of public concern ^25^. As one of the methodological benefits, our workshop methods helped identify who were the influential stakeholders in the formal and communal structures.

### Compensation models to be used for HIS in Zambia

The four compensation models ^20^ were presented and distinguished from each other as in Table 3 the table.

The meeting stressed the need to separate compensation (i.e., wage/reimbursement) from provisions for study related injury (i.e., no-fault insurance). It was concluded that what would work is if Zambia combines the wage and reimbursement compensation models and not the market (demand and supply) or appreciation (token) model. Malawi offered to share their proposed model which could be adapted to the Zambian context. It was also stressed that models that are working for high income settings may not necessarily work in the Zambian context for the same kind of studies. It was suggested that a fair model needed to be put in place that will deal with issues of undue influence and ensure that participants are safe. It was also emphasized that participants needed not to be paid for participating in research but to be compensated for the risk of trial-related injuries. The meeting also emphasized the need for further thought on balancing risk, prevailing economic situations, undue coercion and altruism in HIS.

### Rationale for conduct of HIS

It was noted during the meeting that, there are several reasons which justify HIS in LMIC settings such as Zambia. While capacity to undertake such studies has previously been the main reason to restrict the studies to high income settings, a reassessment was needed to equally justify why such studies may be done in some of high income settings, particularly in the context of vaccine development given that vaccines targeted against infectious diseases are mainly used by LMICs.

The meeting pointed out that principles of justice categorically point to LMIC populations to bear some of the risks and burdens of research as they also stand to benefit from it. It was further argued that, this calls to question implementation of HIS in the high income settings where many of the diseases being studied may not be prevalent. It was argued that there are several factors, including environmental and genetic factors, that may affect how people from LMICs respond to vaccines that are developed and tested exclusively in non-endemic countries. Therefore, it was agreed that it is imperative that HIS trials are conducted in environments and individuals for which the vaccine is intended to be used.

Furthermore, it was argued that findings from vaccine effectiveness reports have overwhelmingly demonstrated that some of the oral vaccines (against rotavirus, polio etc.) ^1^ perform poorly in LMICs compared to Western countries. Multiple reports have alluded to context related differences between populations in high income versus low income settings such as repeated exposure to infectious diseases ^26^ resulting in environmental enteric dysfunction, altered gut microbiome, genetic predispositions etc. as some of the reasons why vaccines perform differently ^27^. The meeting further maintained that, these experiences suggest that research studies need to be undertaken in populations with these contextual factors which are similar for the eventual target populations; thus HIS in LMICs are likely to provide more accurate efficacy and eventual effectiveness data especially on oral vaccines.

The decision to conduct/approve HIS should be based on considerations of the risk-benefit ratio coupled with the ethical principles that ensure of human subject protection and medical ethic of “do no harm”. The meeting agreed that the ethical considerations and regulatory reviews must be paramount while championing local capacity building to develop and conduct HIS relevant to the Zambian people.

### Identifying target participants for HIS

The meeting emphasized that the global ethical principles need to be applied when considering target population for HIS. The rule of thumb must be to target populations best suited to address the principal research questions FIRST. Once the population is identified then other key considerations should be put in place, particularly if the population has elements of “vulnerability” i.e. children, students, prisoners, low-socio-economic populations etc.

It was generally agreed that considerations for target populations must NOT principally avoid vulnerable populations because often those may be the populations affected by problems that require specific research, thus excluding them may only widen the research gap instead of finding specific solutions. Rather, clear justification must be provided why they are chosen and efforts demonstrated on how the vulnerabilities will be mitigated.

The issue of literacy and ability to read and write as a means for better comprehension of the risks involved was discussed at length. It was stressed that while college/university students may be preferred for this reason, there is a need to recognize that there are social-economic vulnerabilities even among this class of society; especially when payment for participation is substantial and may be favourably compared to existing local wages. Examples of students from Western countries were given, in that students usually take part in these studies to make extra money to take care of their everyday needs ^28^.

The meeting concluded that ensuring adequate provision of information, comprehension of the risks involved, and capacity to make a decision to participate entirely of their own free will is most important for the targeted group of individuals. It further emphasized that HIS must not necessarily avoid the poor or uneducated.

### ICF content

The meeting emphasized that the standard principles of informed consent forms (ICF) for trials must also apply to HIS. However, given that typically HIS participants will be healthy and will be deliberately infected, the need to provide very clear information and ensure good participant appreciation of risk cannot be over emphasized.

The meeting argued that, more so, the ICF for HIS must be balanced to provide adequate information given the potential and perceived risks but not make the consenting process laborious.

For example, for highly infectious pathogens, the ICF must be clear that while subjects are free to leave the study at any point, they may be required to remain as “in-patient” until fully treated as a provision for pathogen containment and thus to protect the community in the spirit of public health. It further stated that there was need to be careful not to be seen to override a participants’ right, but to explain in clear terms that having the participant stay at the study facility was for their own good and that of the community they come from. It was emphasized that ensuring that a participant is cleared of infection was not only ethically imperative but ethically justifiable too. Given the various components and nuances, the meeting suggested group consent where information is shared and to move to individual at the written consent stage. It was also argued that audio-visuals and other materials could also be used to ensure that information is clearly communicated and received. It was argued that a participant needed to understand that signing the consent form meant that if they decided to withdraw from the study, they agree to stay in the study facility until a time when the researchers ascertain that they are free of the infection and safe to mingle with the community so as to contain the pathogen.

Nonetheless, after much deliberation, the meeting agreed that “Autonomy is the main principle that justifies HIS”, and the application of informed consent does satisfy the fundamental ethical requirement. 

### Requirements for challenge agent strain(s)

The meeting discussed whether proposed challenge agents should be regulated using good manufacturing practice (GMP) or GMP-like regulations. The regulators indicated that there was need for more time to consider what would be permissible in regulatory guidelines for Zambia and that an engagement meeting with stakeholders, particularly the WHO (through the African regulatory and ethics network), would be important to finalise recommendations that uphold the highest standard of quality assurance and support the local conduct and capacity development for HIS implementation including for the manufacture of challenge agents.

## Conclusions and next steps

The workshop approach (i.e. use of participants empathy maps and journey maps) and less didactic techniques provided an opportunity for rich discussion and brought out issues which would ordinarily have been left out. In addition, the ownership of the whole process of considering what needs to be addressed as the country prepares for the conduct of HIS was enhanced with regulators identifying gaps in the legal and policy frameworks and determining how to address HIS applications and conduct. Bearing in mind the local context, the meeting concluded that scientists needed to leverage public engagement with various stakeholders, as a tool to identify key issues that they need to be considered for the successful implementation of HIS in Zambia.

When discussing guidelines for importation and development of the challenge agent(s) and whether GMP or GMP-like practices were required, it was clear that there is a need to review both provisions and agree on what would be acceptable for Zambia but that all this should be driven by a balance between ensuring quality assurance and promoting local capacity development in the conduct of research.

The workshop also agreed on the need for continuous engagement. Target groups identified included the parliamentary committee on health given their composition and mandate of being law makers; media editors as gatekeepers on information disseminated, published or broadcasted in the press; the use of research champions such as lecturers in tertiary institutions of learning taking advantage of their intellectual independence including their position of high esteem in society; other gate keepers and influential people in target communities as well as the public at large need constant engagement and sensitisation given their hypersensitivity to anything “GMO”.

The discussions around what should be considered adequate compensation for participating in HIS brought to light the fact that while the current formula of arriving at compensation for participating in research in Zambia is based on the wage and reimbursement model, there is need to consider a model which can include other aspects such as “Risk” and “Appreciation”. The meeting also agreed to ensure that when talking about compensation, it is important to ensure that both compensation for participation in HIS (i.e., the burden of participation) and cover for injury due to participation in HIS are considered.

Lastly, there was consensus on the need to review the current guidelines and provisions for the conduct of HIS including those under development by the World Health Organisation (WHO) to ensure that gaps in Zambian provisions are covered and updated.

The engagement workshop on the conduct of HIS in Zambia successfully introduced these types of studies to regulators, ethicists, scientists and other key stakeholders as well as identified issues to be considered in view of setting up HIS in Zambia. Through the platform that was created: (i) the review of HIS guidelines and legal frameworks to both address local considerations and align with international ones; (ii) review of the GMP and GMP-like requirements and propose what might be acceptable for Zambia; as well as (iii) the development of an engagement strategy for conduct of HIS in Zambia will be ongoing. We summarize the discussions in Table 4.

**Table 4.** Summary of key issues raised during the workshop.

Methodology	Issues raised
**Didactic sessions**	** Justification for conducting HIS** • Prior to having regulatory guidelines specifically on CHIM, Kenya applied a do no harm principle and other ethical principles as applied to HIS elsewhere • Individual Autonomy main principle justifying human infection studies • The rationale for doing HIS should be based on the Risk-Benefit ratio • Target population/participants should be drawn from the population at risk of disease • Nurture local research and development of scientific products • Examine the regulations under which HIS may be undertaken in Zambia (NHRA/NBA/ZAMRA) **Challenge Agents** • Choice of challenge/pathogen must be based on the reversibility of infections • Importation and development of challenge agent ◦ ZAMRA: (i) Check if challenge agent can be imported under the importation of vaccines as per WHO guidelines; (ii) expedite current legal review to have HIS and challenge agent importation incorporated; (iii) if the two options fail, seek statutory Instrument through the Ministry of Health to provide for importation of challenge agents ◦ NBA: currently no provision for importation of GMO or challenge strain for contained use • Requirement for GMP/GMP-like challenge agents ◦ First use in setting be GMP product ◦ No harmonised/standardised way (even in the West – UK) on challenge agent preparation • Public awareness is critical given the hypersensitivity around GMOs (i.e. public and private universities through their lecturers/ researchers, advocates, parliamentary committee on health) • **Community engagement** and incorporation of nested social science studies in all HIS • We need consider aspects of social harm (e.g., contraceptive use while in study, conjugal rights/visits while in residence for HIS) ** Compensation** • Need to determine what constitutes ‘fair compensation’ and a model to arrive at the appropriate figure • Compensation determination model (i.e. risk vs wage based). ◦ Malawi has a proposed model which can be looked at by Zambia and adapted ◦ Need to separate compensation (i.e. wage/reimbursement) and provision for study related injury (i.e. no-fault insurance)
**Group discussions**	• Target population/participants for consideration ◦ Student/college level for initial implementation for adult studies ◦ Study staff participation to emphasise and enforce trust • HIS less risky in comparison to traditional Phase I (first-in-man) studies • Potential Tax payments on compensation – requirement to engage for local context • Media engagement ◦ When is right time to engage – need to consider editors? ◦ Need to have a response in hand in case of bad press attention ◦ Need buy-in from regulators on response to bad press • Confidentiality – protection of study participant rights • When using live attenuated vaccines as challenge agents, does dose vary? • Consent – who is empowered to give full consent? • Compensation for infants/children (under 14 years) • Privacy and comfort in residence • Compensation and access to care agreed at consenting process • Right to withdraw – need to have cleared infection and treated before exiting the study/in-patient • Burden – infringement/inconveniences e.g. time, travel for procedures • Risk – bodily harm • Consideration of previous experience with challenge agent • ICF needs to be clear regarding procedure for withdraw, in Malawi ICF is a legal document and is in the constitution • Perceived benefits of HIS included; fewer people exposed to harm, quick answer to research questions, product/intervention available to people at risk, knowledge about disease treatment and management • Safety in conduct of HIS can be assured by; adequate infrastructure (i.e. safe storage and use of IP, emergency resuscitation and evacuation) trained and qualified personnel, GCP compliance, extensive screening for inclusion criteria, DSMB, reversibility of disease, HIS oversight by REGs and ethics, community sensitisation • Consider if the right to withdraw is equal to right to refuse treatment and that if faced with a withdraw from study: ◦ “Public health need” should be considered before allowing withdraw ◦ Follow up post HIS participation critical to ensure safety

*Note: List of abbreviations **CIDRZ      Centre for infectious disease research in Zambia** **CHIM      Controlled Human Infection Model** **DSMB      Data Safety Monitoring Board** **GCP      Good Clinical Practice** **GMO      Genetic Modified Organism** **GMP      Good Manufacturing Practice** **HIS      Human Infection Studies** **ICF      Informed Consent Form** **IP        Investigational Product** **KEMRI      Kenya Medical Research Institute** **LMICs      Low and Middle Income Countries** **NBA      National Biosafety Authority** **NHRA      National Health Research Authority** **REGs      Regulatory authorities** **WHO      World Health Organization** **ZAMRA      Zambia Medicines Regulatory Authority**

## Data availability

### Underlying data

No data is associated with this article
