# Supplementary material for: Engagement of ethics and regulatory authorities on human infection studies: Proceedings of an engagement workshop in Zambia
Source: Wellcome Open Res. 2021 Sep 14;6:31. Originally published 2021 Feb 12. [Version 2] doi: 10.12688/wellcomeopenres.16432.2 (PMC7993625; doi:10.12688/wellcomeopenres.16432.2)
Supplement: Peer review history

# Peer review: Reviewer response for version 1

**DOI:** 10.21956/wellcomeopenres.18083.r42969

**Published:** 2021-03-23

**Authors:** Lucinda Manda-Taylor

**Affiliations:** 1 School of Public Health and Family Medicine, College of Medicine, University of Malawi, Blantyre, Malawi

The authors have done a good job and presenting the results of the stakeholder engagement workshop in Zambia. The workshop included a variety of individuals who could contribute to the discussion on the establishment and conduct of HIS research in Zambia. Key topical issues covered are the need to do this research in LMICs like Zambia, the review of the legal and regulatory environment, and key ethical issues that need to be considered in the conduct of HIS research. The report covers these aspects well, but there are a few areas that require clarity, amendments and considerations for inclusion.

1. In the abstract, the authors should add the word "officials" to the sentence, "We engaged bioethicists, regulatory authority officials and scientists in Zambia, other African countries and the funder".
2. In the last paragraph of the introduction, the authors should also include Vietnam as a country where similar stakeholder engagement workshops have been conducted  - Kestelyn E, Le Phuong C, Ilo Van Nuil J *et al.* Expert voices and equal partnerships: establishing Controlled Human Infection Models (CHIMs) in Vietnam [version 1; peer review: 3 approved]. *Wellcome Open Res* 2019, **4**:143 ( https://doi.org/10.12688/wellcomeopenres.15337.1). In this manuscript, the authors define the actual questions that structured the workshop and it is worth including this to also demonstrate how their workshop followed a similar structure particularly in exploring ethical issues around risk, compensation, consent and public engagement.
3. For clarity, the authors should rephrase the sentence Under **Summary of the considerations for HIS in Zambia **to read as follows: Twenty-nine participants were drawn from four different countries: Malawi, Kenya, UK, and Zambia. These participants were drawn from 4 specialisations, including ethics, community engagement, social science, funder, regulatory officials and clinical trial scientists, as shown in Table 2.
4. Under the Legal Provisions and Regulatory Guidelines, were there any suggestions offered on how the process to amend the NHRA Act should be done? If yes, a statement on the process would be valuable to include.
5. On Compensation, did the stakeholders agree on when and how compensation would be provided to participants? Would it be daily or would it be given at the end of the study? How would compensation work if an individual joins the study and decides to leave during the period under quarantine?
6. Were any specific recommendations provided by stakeholders on what key ethical considerations must be considered when researching on children?
7. In the presentation of the report, it would be worthwhile for the authors to compare their findings from other stakeholder workshops that have dealt with the same topics or issues in order to demonstrate how perhaps there s a consensus of views from other engagement workshops on, for example, compensation, the absence of clear regulatory and legal guidance, etc. By weaving finding from across other LMICs that are exploring or have explored the same issues this report could have a section before the conclusion and next steps headed, Summary, that highlights common concerns, challenges in conducting HIS in LMICs while maintaining that the specific findings of this report will inform the implementation of HIS in Zambia leading to conclusions and next steps.

Does the article adequately reference differing views and opinions?

Partly

Are all factual statements correct, and are statements and arguments made adequately supported by citations?

Yes

Is the Open Letter written in accessible language?

Yes

Where applicable, are recommendations and next steps explained clearly for others to follow?

Yes

Is the rationale for the Open Letter provided in sufficient detail?

Yes

Reviewer Expertise:

Bioethics and Social Science

I confirm that I have read this submission and believe that I have an appropriate level of expertise to confirm that it is of an acceptable scientific standard.

# Peer review: Reviewer response for version 1

**DOI:** 10.21956/wellcomeopenres.18083.r42613

**Published:** 2021-03-08

**Authors:** Jeffrey D'Souza

**Affiliations:** 1 Institute on Ethics & Policy for Innovation, Department of Philosophy, McMaster University, Hamilton, Canada

In “Engagement of ethics and regulatory authorities on human infection studies: Proceedings of an engagement workshop in Zambia,” Kunda-Ng’andu et. al. provide an overview of a workshop held in Siavonga, Zambia on conducting human infection studies (HIS) in low and middle-income countries. In this Open Letter, the authors provide a summary of the central ethical, legal, and regulatory issues raised at similar workshops held in Vietnam, Malawi, Kenya, Uganda, and India, and highlight what “stood out” at the Zambian meeting and stands out in the Zambian context. The findings presented in the aforementioned manuscript are significant and may be used to help inform future engagement workshops on HIS, as well as policy and practice in Zambia, and other geographies and countries.

 The following seven queries are intended to draw greater insight from the knowledge exchanged during the workshop, clarify the views presented in this manuscript, and bolster the impact this paper makes on this niche area of research and policy.   

1. The authors note that one of the main reasons for conducting HIS is due to the health disparity that exists among children in high-income countries and low and middle-income countries. However, other than noting that children may be understood as one vulnerable group among others, the manuscript does not mention any particular regulatory or ethical issues pertaining to conducting HIS with children. Did the participants at the engagement workshop explore potentially conducting HIS involving children?  If the challenges related to conducting HIS involving children were discussed, it is important that these discussions are shared in this paper to help inform an important and timely debate.
2. On page 6, the authors note that “unlike in India, we discussed the need to explore how HIC fits in existing regulatory frameworks” as opposed to developing new regulatory and ethical frameworks in Zambia. Are the authors able to explain why and how participants arrived at this decision? Such insight may prove to be helpful for other stakeholders and policy makers contemplating, and wrestling with this important decision.
3. The proceedings as presented appear to be favorable toward conducting HIS in Zambia. Was there any direct or indirect opposition to this position? It is important that the proceedings reflect dissenting views (if any were present) to provide an accurate, and wholistic portrayal of views presented at the workshop.  
4. When did the engagement workshop take place in Siavonga? The date will help situate the proceedings presented, especially if there was a significant delay between the meeting and publication date.  
5. On page 9, the authors refer to “global ethical principles.” Here, it would be helpful if the authors clarify what particular principles they are referring to in this context.
6. Are the authors able to share the list of pre-read materials that were shared with participants in the workshop? This information may help inform the design of future engagement workshops on HIS as well as help to situate the findings of the workshop.
7. Lastly, while the paper is, generally speaking, clear, and well-written, the paper should be further proof-read as there remain a few small typos throughout (e.g., unnecessary capitalization of “FIRST” on page 9.) Also, it would be helpful to the reader to use one acronym throughout the paper instead of switching between “CHIM”, “HIC”, and “HIS” to refer to human infection studies. 

In summary, Kunda-Ng’andu *et al.* provide key insights into stakeholders’ views regarding conducting HIS in Zambia. This Open Letter publication serves as an important guiding piece in the development of future engagement workshops on HIS, as well policy and practice in Zambia, and other geographies and countries.

Does the article adequately reference differing views and opinions?

Yes

Are all factual statements correct, and are statements and arguments made adequately supported by citations?

Yes

Is the Open Letter written in accessible language?

Yes

Where applicable, are recommendations and next steps explained clearly for others to follow?

Yes

Is the rationale for the Open Letter provided in sufficient detail?

Yes

Reviewer Expertise:

Global Health Ethics; Ethics of Clinical Trials; Research Ethics; Global Bioethics; and Global Justice.

I confirm that I have read this submission and believe that I have an appropriate level of expertise to confirm that it is of an acceptable scientific standard.
